# Water as Source of *Francisella tularensis* Infection in Humans, Turkey

**DOI:** 10.3201/eid2112.150634

**Published:** 2015-12

**Authors:** Selcuk Kilic, Dawn N. Birdsell, Alper Karagöz, Bekir Çelebi, Zekiye Bakkaloglu, Muzaffer Arikan, Jason W. Sahl, Cedar Mitchell, Andrew Rivera, Sara Maltinsky, Paul Keim, Duran Üstek, Rıza Durmaz, David M. Wagner

**Affiliations:** Public Health Institution of Turkey, Ankara, Turkey (S. Kilic, A. Karagöz, B. Çelebi, Z. Bakkaloglu, R. Durmaz);; Northern Arizona University, Flagstaff, Arizona, USA (D.N. Birdsell, J.W. Sahl, C. Mitchell, A. Rivera, S. Maltinsky, P. Keim, D.M. Wagner);; Istanbul University, Istanbul, Turkey (M. Arikan);; Medipol University, Istanbul (D. Üstek)

**Keywords:** *Francisella tularensis* infections, *Francisella tularensis* subsp. *holarctica*, phylogeography, SNP, canSNP, Turkey, bacteria, tularemia, lineage, zoonoses

## Abstract

*Francisella tularensis* DNA extractions and isolates from the environment and humans were genetically characterized to elucidate environmental sources that cause human tularemia in Turkey. Extensive genetic diversity consistent with genotypes from human outbreaks was identified in environmental samples and confirmed water as a source of human tularemia in Turkey.

Tularemia is a disease caused primarily by 2 subspecies of *Francisella tularensis*: *F. tularensis* subsp. *tularensis*, which is restricted to North America; and *F. tularensis* subsp. *holarctica*, which is found widely throughout the northern hemisphere but is the only subspecies in most of Eurasia (*1*). Through whole-genome sequencing and canonical single-nucleotide polymorphism (canSNP) genotyping, *F. tularensis* subsp. *holarctica* has been divided into 4 major genetic groups (B.4, B.6, B.12, and B.16) consisting of multiple subgroups (Figure 1) (*1*–*3*). Geographic distribution of these subgroups in Europe, Japan, and the USA are well described (*1*–*3*).

**Figure 1:**
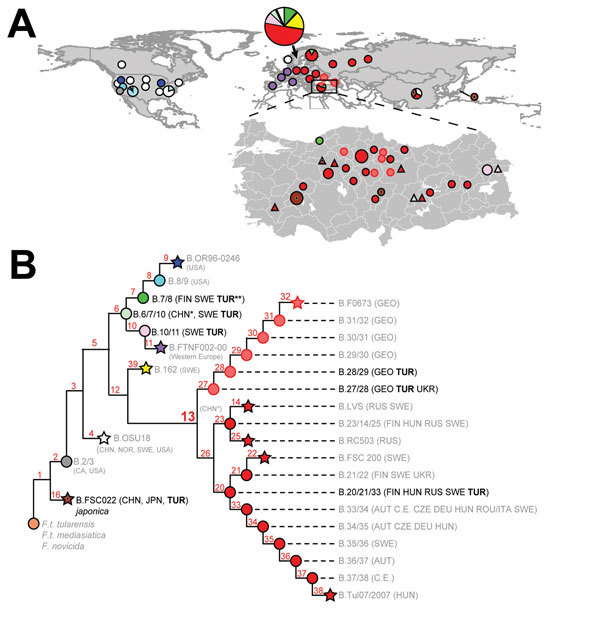
Phylogeography of *Francisella tularensis* subsp. *holarctica*. A) Global distribution of known phylogenetic groups determined on the basis of previous studies (*2**–**4*); enlarged map of Turkey shows locations of phylogenetic groups identified among the 40 samples positive for *F. tularensis* examined in this and previous studies (*5*). Circle size indicates number of samples (small circles, 1–3; medium circles, 4–6; large circles, 7–9). Colors of circles (human samples) and triangles (environmental samples) represent the phylogenetic subgroups to which these samples were assigned (see panel B). Subgroup B.16 (biovar *japonica*) is represented by the dot inside the brown circles and triangles. B) Phylogenetic tree for *F. tularensis* subsp. *holarctica* constructed on the basis of current canonical single-nucleotide polymorphism genotyping. Red numbers indicate nomenclature of canonical single-nucleotide polymorphism groups. Terminal subgroups representing sequenced strains are shown as stars, and intervening nodes representing collapsed branches are indicated by circles. Countries of origin for samples assigned to relevant phylogenetic groups are as follows: AUT, Austria; CE, central Europe, unknown country; CHN, China; CZE, Czech Republic; DEU, Germany; FIN, Finland; GEO, Georgia; HUN, Hungary; ITA, Italy; NOR Norway; ROU, Romania; RUS, Russia; SWE, Sweden; TUR, Turkey; UKR, Ukraine; USA, United States. CHN* indicates approximate phylogenetic placement because of a lack of resolved information on single-nucleotide polymorphisms (*4*). TUR** indicates identification from a previous study (*5*).

The phylogeography of *F. tularensis* in Asia is poorly understood because of undersampling in many regions, but recent studies have revealed new insights. A report has described rich phylogenetic diversity of the bacterium in China (*4*), including the rare B.16 group (biovar *japonica*). Previously, B.16 was known only in Japan (*1*) and Turkey (*6*). Sweden reportedly has the highest overall phylogenetic diversity among regions worldwide (*2*).

In Turkey, tularemia cases in humans have increased since 2009 (*7*), but little is known about environmental sources. Tularemia was first reported in Turkey in 1936 and then was sporadically reported for several decades (*7*). After improved surveillance, the number of tularemia cases increased in the 1980s and led to registration of tularemia as a reportable disease in 2004 (*7**,**8*). Incidence has continued to increase since then (*7*), and tularemia is now considered a reemerging zoonotic disease in Turkey.

Patients with oropharyngeal signs and symptoms account for ≈90% of tularemia cases in Turkey (*8*), and cases emerge seasonally from August–March (*7*). Seasonality of incidence of cases is presumably associated with consumption of contaminated water (*9*), but confirming sources is difficult. Reports of confirmation of *F. tularensis* from water samples by PCR (*10*) or culture (*6*) are rare, and definitive studies that link water to tularemia in humans are lacking. How water sources become seasonally contaminated is also unknown, but contamination could be caused by rodents. Recently, *F. tularensis* was confirmed by PCR from 2 mice captured in Thrace (*11*), but in Turkey, confirmation has not been obtained from ticks or mosquitoes, which are known vectors of *F. tularensis* (*1*,*4*).

Genetic characterization of clinical samples from tularemia outbreaks in Turkey in 2011 showed that multiple phylogenetic groups cause disease in multiple regions across Turkey (*5*); however, no environmental samples were assessed in that study. We report our findings from genetically characterized samples positive for *F. tularensis* from environmental and human sources located in multiple active tularemia areas in Turkey. Our results provide new insights into *F. tularensis* transmission from environmental sources to humans.

## The Study

To examine environmental reservoirs that could be possible sources for human infections, during 2010–2013, we sampled water sources and rodent populations from suspected sites where transmission of *F. tularensis* infection could occur in Turkey. To survey and compare phylogenetic diversity of environmental samples and clinical samples, we examined 33 clinical samples of mostly oropharyngeal tularemia cases from approximately the same sites where environmental samples were collected. DNA was extracted (DNeasy Blood & Tissue Kit, QIAGEN GmbH, Hilden, Germany) from 6 water, 1 rodent spleen, and 33 human samples (Technical Appendix Table 1).

The extractions were confirmed *F. tularensis*–positive by using PCR and targeting the *tul*4 gene (*12*). Analysis by using 21 published canSNP assays, as previously described (*5*), assigned these samples to 3 major phylogenetic groups and distinct subgroups: B.16 (n = 11); B.6 (2 subgroups: B.6/7/10, n = 1; and B.10/11, n = 6); and B.13 (2 subgroups: B.27, n = 5; and B.20/21/33, n = 17) (Figure 1; Technical Appendix Table 1). Of the subgroups, 3 were previously unknown in Turkey: B.6/7/10, B.10/11, and B.16. The 7 environmental samples collected included most of the known phylogenetic diversity in Turkey and represented the 3 major groups: B16, B6 (B.6/7/10 and B.10/11), and B.13 (the group previously known to be in Turkey). Of the subgroups identified, all but B.6/7/10 were also found in the human samples.

To determine detailed associations between environmental and human clinical samples, we examined the genetic diversity among these samples by using multilocus variable number of tandem repeats analysis (MLVA) (*13*). All samples contained a single MLVA genotype (Technical Appendix Figure, panels A–C); no mixed allele calls were observed at any of the examined variable number of tandem-repeats loci. Three different environmental samples (F0922, F0910, and F0916) had canSNP and MLVA genotypes that were identical to those of clinical samples (Technical Appendix Table 1). In 2 instances (F0910 and F0916), the environmental sample and its respective genetically identical clinical sample(s) were recovered from different geographic regions, resulting in identical genotypes being found in different localities and suggesting that close genotypes are dispersed widely in Turkey. One environmental sample (F0922) had genetic, geographic, and temporal data (Technical Appendix Figure, panel A) concordant with data from human samples. This water sample shared identical canSNP and MLVA genotypes with 5 clinical samples recovered 2 weeks previously at the same locality, strongly suggesting that the human cases are linked with this infected water source.

The genetic characterization of *F. tularensis* from environmental sources provides insights into transmission of tularemia from the environment to humans, but little is known about how water is contaminated. The seasonal nature of human outbreaks suggests that water sources are not constant reservoirs but rather are contaminated by another source. Rodents were identified as reservoirs (21% tularemia positive) in Bulgaria, where mainly oropharyngeal tularemia is endemic (*14*). We found a rodent sample (F0910) with canSNP and MLVA genotypes identical to an oropharyngeal clinical sample (F0898) (Technical Appendix Table 1), a finding consistent with water contamination that originates from animal sources. However, the converse is also possible: animals could become infected by contaminated water.

Analysis of the 7 environmental *F. tularensis* subsp. *holarctica* samples from Turkey revealed extensive phylogenetic diversity that represents most known major groups in the world. Three of the 4 major *F. tularensis* subsp. *holarctica* phylogenetic groups (B.4, B.6, B.12, and B.16) are found in Turkey, including the highly basal B.16 group (biovar *japonica*) (Figure 1). This finding indicates that no single phylogenetic type is dominant in Turkey, unlike in Western Europe (*3*). Diversity was also represented in the clinical samples, suggesting that all major groups have similar capacities to cause disease, as other studies have suggested (*15*).

**Figure 2:**
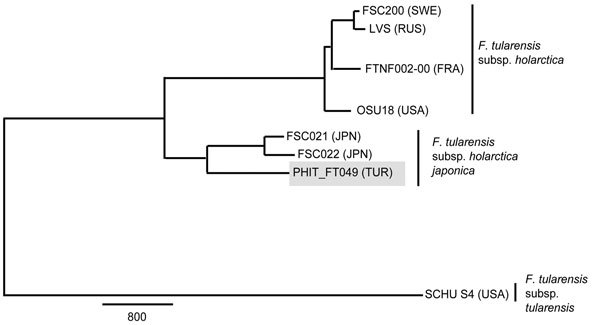
Maximum-parsimony phylogeny constructed by using 10,443 putative single-nucleotide polymorphisms discovered from whole-genome sequences of 8 *Francisella tularensis* strains. Gray shading indicates the B.16 (biovar *japonica*) strain from Turkey (PHIT_FT049). Detailed methods are described in the Technical Appendix. Reference strains were retrieved from GenBank (Technical Appendix Table 2). Countries of origin are indicated as follows: FRA, France; JPN, Japan; RUS, Russia; SWE, Sweden; TUR, Turkey; USA, United States. Scale bar indicates single-nucleotide polymorphisms.

To gain insights into the evolutionary origin of the B.16 group, we examined the phylogenetic relationships among 3 published B.16 strains: 1 from Turkey (PHIT-FT049) (*6*) and 2 from Japan (FSC021 and FSC022) (GenBank accession nos. CP007148.1, SRX147922, and DS264138.1, respectively; Figure 2). We generated a global core-genome SNP phylogeny (Technical Appendix) for these 3 B.16 strains and 5 strains from other groups (Technical Appendix Table 2). As expected, PHIT-FT049 clusters with the Japanese B.16 strains from Japan and shares 448 putative SNPs; however, it is also distinct from the 2 strains from Japan, which together share 640 putative SNPs (Figure 2). The distinctiveness of the B.16 strain from Turkey strongly suggests that it has an evolutionary history different from that of the Japanese strains. The MLVA phylogeny of B.16 strains (Technical Appendix Table 1) reveals greater diversity among the 8 strains from Japan than among the 8 strains from Turkey. These data show that the B.16 strains from Turkey and Japan are highly distinct, and the greater diversity in strains from Japan supports the possibility that the place of ancestral origin of the B.16 group is Asia.

## Conclusions

Phylogenetically diverse strains of *F. tularensis* subsp. *holarctica* are environmentally established in Turkey and cause human disease. The strains in Turkey now include many phylogenetic groups previously found only in Scandinavia or Asia. 

**Technical Appendix.** Detailed methods for constructing the phylogeny in Figure 2, detailed information about the samples and reference strains examined in this study, and a phylogeny constructed with data from a multilocus variable number of tandem repeats analysis.
