# Supplementary material for: Water as Source of Francisella tularensis Infection in Humans, Turkey
Source: Emerg Infect Dis. 2015 Dec;21(12):2213–6. doi: 10.3201/eid2112.150634 (PMC4672436; doi:10.3201/eid2112.150634)
Supplement: Supplementary file 1 — Technical Appendix. Detailed methods for constructing the phylogeny in Figure 2, detailed information about the samples and reference strains examined in this study, and a phylogeny constructed with data from a multilocus variable number of tandem repeats analysis. [file 15-0634-Techapp-s1.pdf]

Article DOI: http://dx.doi.org/10.3201/eid2112.150634

Water as Source of *Francisella tularensis* Infection in Humans, Turkey

Technical Appendix

Details of Samples and Reference Strains in This Study

Detailed Methods for Constructing the Phylogeny in Figure 2

Published reference genome assemblies (Technical Appendix Table 2) were downloaded from GenBank (*1*). Assemblies were aligned against the reference genome, *F. tularensis* subsp. *holarctica* OSU18, by using MUMer (*2*). The reference genome was also aligned against itself; regions that aligned >1 time represent duplication events and were filtered from downstream analyses. Single-nucleotide polymorphisms compared with the reference were concatenated, and a maximum-parsimony phylogeny (Figure 2) was inferred on a concatenation of ≈15,000 single-nucleotide polymorphisms by using Phangorn (*3*).

Technical Appendix Table 1. Details of samples from study of *Francisella tularensis* infection, Turkey

Original ID*	NAU ID†	County/Region	City	Source	Sample Type	Date	SNP subgroup‡
PHIT-FT049, F283§	F0915	Central Anatolia	Ankara	Water	DNA extract from isolate cultured from water	3/12/2012	B.16
F059	F0892	Aegean	Afyonkarahisar	Human lymph node	DNA extract from clinical sample	2/19/2010	B.16
F060	F0893	Aegean	Afyonkarahisar	Human lymph node	DNA extract from clinical sample	2/19/2010	B.16
F062	F0894	Aegean	Afyonkarahisar	Human lymph node	DNA extract from clinical sample	2/19/2010	B.16
F063	F0895	Aegean	Afyonkarahisar	Human lymph node	DNA extract from clinical sample	2/19/2010	B.16
F064	F0896	Aegean	Afyonkarahisar	Human lymph node	DNA extract from clinical sample	2/19/2010	B.16
F069	F0899	Central Anatolia	Kayseri	Human lymph node	DNA extract from clinical sample	2/24/2010	B.16
F071	F0900	Aegean	Afyonkarahisar	Human lymph node	DNA extract from clinical sample	2/26/2010	B.16
F072	F0901	Aegean	Afyonkarahisar	Human lymph node	DNA extract from clinical sample	2/26/2010	B.16
F085	F0902	Aegean	Afyonkarahisar	Human lymph node	DNA extract from clinical sample	3/19/2010	B.16
F272	F0912	Central Anatolia	Kayseri	Human lymph node	DNA extract from clinical sample	2/8/2012	B.16
F015	F0884	Central Anatolia	Çankırı	Human throat swab	DNA extract from isolate cultured from human	1/8/2010	B.28/29
F026	F0885	Black Sea	Amasya	Human throat swab	DNA extract from isolate cultured from human	1/18/2010	B.28/29
F217	F0907	Eastern Anatolia	Sivas	Human throat swab	DNA extract from isolate cultured from human	4/12/2011	B.28/29
F043	F0890	Central Anatolia	Yozgat	Human lymph node	DNA extract from isolate cultured	1/27/2010	B.28/29

Original ID*	NAU ID†	County/Region	City	Source	Sample Type	Date	SNP subgroup‡
					from human		
F039	F0889	Black Sea	Tokat	Human lymph node	DNA extract from isolate cultured from human	1/22/2010	B.27/28
F303-s291, F291	F0923	Aegean	Denizli	Water	DNA extract from isolate cultured from water	12/12/2013	B.20/21/33
F049	F0891	Central Anatolia	Kirsehir	Human throat swab	DNA extract from isolate cultured from human	2/8/2010	B.20/21/33
F065	F0897	Black Sea	Tokat	Human lymph node	DNA extract from isolate cultured from human	2/19/2010	B.20/21/33
F067	F0898	Central Anatolia	Kirikkale	Human conjunctival swab	DNA extract from isolate cultured from human	2/8/2010	B.20/21/33
F236	F0908	Black Sea	Ordu	Human throat swab	DNA extract from isolate cultured from human	4/12/2011	B.20/21/33
F282	F0914	Central Anatolia	Sivas	Water	DNA extract from isolate cultured from water	3/6/2012	B.20/21/33
F244	F0910	Central Anatolia	Ankara	Rodent/spleen	DNA extract from isolate cultured from rodent	11/14/2011	B.20/21/33
F037	F0888	Central Anatolia	Corum	Human lymph node	DNA extract from isolate cultured from human	1/20/2010	B.20/21/33
F027	F0886	Black Sea	Amasya	Human throat swab	DNA extract from isolate cultured from human	1/18/2010	B.20/21/33
F033	F0887	Black Sea	Amasya	Human throat swab	DNA extract from isolate cultured from human	1/18/2010	B.20/21/33
F237	F0909	Eastern Anatolia	Elazig	Human lymph node	DNA extract from isolate cultured from human	8/16/2011	B.20/21/33
F091	F0903	Central Anatolia	Yozgat	Human lymph node	DNA extract from isolate cultured from human	4/13/2010	B.20/21/33
F159	F0904	Central Anatolia	Kayseri	Human blood	DNA extract from isolate cultured from human	1/29/2011	B.20/21/33
F285	F0916	Eastern Anatolia	Malatya	Water	DNA extract from isolate cultured from water	4/5/2012	B.20/21/33
F163	F0905	Central Anatolia	Kayseri	Blood	DNA extract from isolate cultured from human	2/2/2011	B.20/21/33
F252	F0911	Eastern Anatolia	Mus	Human throat swab	DNA extract from isolate cultured from human	12/15/2011	B.20/21/33
F176	F0906	Eastern Anatolia	Bingöl	Human throat swab	DNA extract from isolate cultured from human	2/21/2011	B.20/21/33
F278	F0913	Eastern Anatolia	Malatya	Water	DNA extract from isolate cultured from water	2/21/2012	B.6/7/10
F293	F0917	Eastern Anatolia	Agri	Human throat swab	DNA extract from isolate cultured from human	2/13/2013	B.10/11
F294	F0918	Eastern Anatolia	Agri	Human throat swab	DNA extract from isolate cultured from human	2/14/2013	B.10/11
F295	F0919	Eastern Anatolia	Agri	Human throat swab	DNA extract from isolate cultured from human	2/14/2013	B.10/11
F297	F0920	Eastern Anatolia	Agri	Human throat swab	DNA extract from isolate cultured from human	2/14/2013	B.10/11
F292	F0921	Eastern Anatolia	Agri	Human throat swab	DNA extract from isolate cultured from human	2/14/2013	B.10/11

Original ID*	NAU ID†	County/Region	City	Source	Sample Type	Date	SNP subgroup‡
F296	F0922	Eastern Anatolia	Agri	Water	DNA extract from isolate cultured from water	2/28/2013	B.10/11

*Strain identification (ID) from Northern Arizona University, Flagstaff, AZ, USA.
†Strain ID from Public Health Institution of Turkey Microbiology Reference Laboratories.
‡Subgroup (*4*).
§Published as GenBank accession no. CP007148.1 (National Center for Biotechnology Information, Bethesda, MD, USA).

Technical Appendix Table 2. Reference strains used in study of *Francisella tularensis* infection, Turkey

Reference strain	WGS accession no.
FSC022	AAYD00000000.1
FSC021	SRX147922
PHIT_FT049	CP007148.1
FSC200	NC_019551.1
LVS	NC_007880.1
FTNF002-00	NC_009749.1
OSU18	NC_008369.1
Schu S4	NC_006570.2

*WGS, whole genome shotgun sequencing data, National Center for Biotechnology Information, Bethesda, MD, USA.

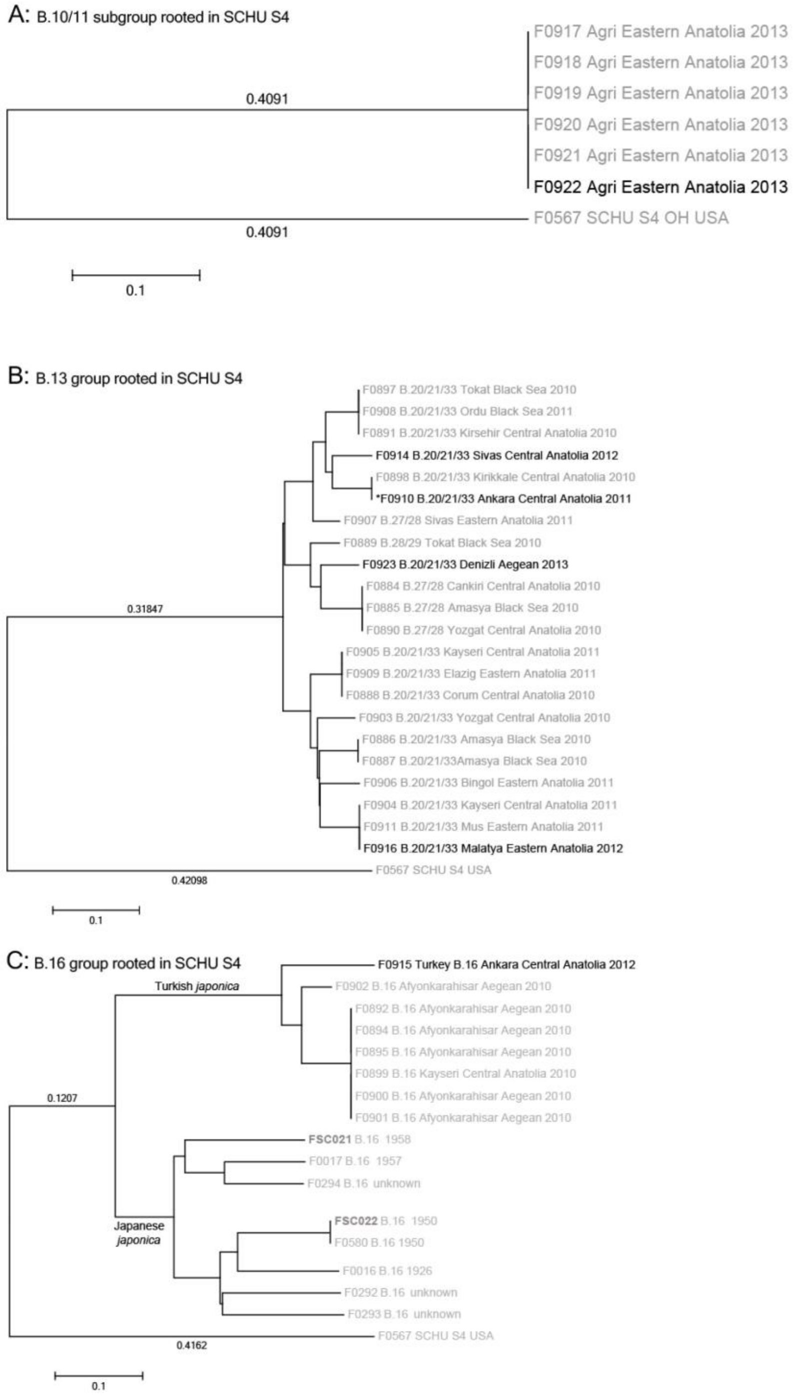

Technical Appendix Figure. Multilocus variable number of tandem repeats analysis (MLVA) trees constructed on the basis of distance matrix. Environmental samples (water and * rodent source) are indicated with bolded font. Scale bar indicates genetic distance. A) MLVA phylogeny for the B.10/11 group, which is rooted by using the SCHU S4 strain published in GenBank. B) MLVA phylogeny for the B.13 group, which is rooted by using the SCHU S4 strain. C) MLVA phylogeny for the B.16 group, which is rooted by using the SCHU S4 strain.

References

1. Benson DA, Karsch-Mizrachi I, Clark K, Lipman DJ, Ostell J, Sayers EW. GenBank. Nucleic Acids Res. 2012;40:D48–53. http://dx.doi.org/10.1093/nar/gkr1202

2. Delcher AL, Salzberg SL, Phillippy AM. Using MUMmer to identify similar regions in large sequence sets. Curr Protoc Bioinformatics. 2003;Chapter 10:Unit 10.3. http://dx.doi.org/10.1002/0471250953.bi1003s00 **PMID: 18428693**

3. Schliep KP. phangorn: phylogenetic analysis in R. Bioinformatics. 2011;27:592–3. http://dx.doi.org/10.1093/bioinformatics/btq706

4. Gyuranecz M, Birdsell DN, Splettstoesser W, Seibold E, Beckstrom-Sternberg SM, Makrai L, et al. Phylogeography of *Francisella tularensis* subsp. holarctica, Europe. Emerg Infect Dis. 2012;18:290–3. http://dx.doi.org/10.3201/eid1802.111305
